# Unveiling early-life microbial colonization profile through characterizing low-biomass maternal-infant microbiomes by 2bRAD-M

**DOI:** 10.3389/fmicb.2025.1521108

**Published:** 2025-01-24

**Authors:** Shuwen Hou, Yuesong Jiang, Feng Zhang, Tianfan Cheng, Dan Zhao, Jilong Yao, Ping Wen, Lijian Jin, Shi Huang

**Affiliations:** ^1^Division of Applied Oral Sciences and Community Dental Care, Faculty of Dentistry, The University of Hong Kong, Hong Kong SAR, China; ^2^Division of Stomatology, Shenzhen Maternity and Child Healthcare Hospital, Southern Medical University, Shenzhen, China; ^3^Division of Periodontology and Implant Dentistry, Faculty of Dentistry, The University of Hong Kong, Hong Kong SAR, China; ^4^Department of Implant Dentistry, Beijing Stomatological Hospital, Capital Medical University, Beijing, China; ^5^Division of Obstetrics and Gynecology, Shenzhen Maternity and Child Healthcare Hospital, Southern Medical University, Shenzhen, China; ^6^Institute of Maternal and Child Medicine, Shenzhen Maternity and Child Healthcare Hospital, Southern Medical University, Shenzhen, China; ^7^Shenzhen Key Laboratory of Maternal and Child Health and Diseases, Shenzhen, China

**Keywords:** breast milk, meconium, low-biomass microbiota, 2bRAD-M, early-life microbiome

## Abstract

**Introduction:**

The microbial composition of human breast milk and infant meconium offers critical insights into the early microbial colonization profile, and it greatly contributes to the infant’s immune system and long-term health outcomes. However, analyzing these samples often faces technical challenges and limitations of low-resolution using conventional approaches due to their low microbial biomass.

**Methods:**

Here, we employed the type IIB restriction enzymes site-associated DNA sequencing for microbiome (2bRAD-M) as a reduced metagenomics method to address these issues and profile species-level microbial composition. We collected breast milk samples, maternal feces, and infant meconium, comparing the results from 2bRAD-M with those from both commonly used 16S rRNA amplicon sequencing and the gold-standard whole metagenomics sequencing (WMS).

**Results:**

The accuracy and robustness of 2bRAD-M were demonstrated through its consistently high correlation of microbial individual abundance and low whole-community-level distance with the paired WMS samples. Moreover, 2bRAD-M enabled us to identify clinical variables associated with infant microbiota variations and significant changes in microbial diversity across different lactation stages of breast milk.

**Discussion:**

This study underscores the importance of employing 2bRAD-M in future large-scale and longitudinal studies on maternal and infant microbiomes, thereby enhancing our understanding of microbial colonization in early life stages and demonstrating further translational potential.

## Introduction

Human microbiome in mothers and infants provides valuable insights into early microbial colonization, which plays a significant role in the development of the infant’s immune system and overall health (Koren et al., 2023; Notarbartolo et al., 2022). Among various body sites of the mother, breast milk is found to contribute profoundly to maternal vertical seeding of infant microbiome even 1 year postnatally (Pannaraj et al., 2017; Wang et al., 2020; Ferretti et al., 2018). However, significant challenges exist in studying the microbiomes in breast milk and infant meconium samples due to the high levels of host contamination and low microbial biomass issues (Oikonomou et al., 2020).

Human milk possesses a relatively low microbial load, with approximately 10^5^ to 10^6^ microbial cells per milliliter of milk (Notarbartolo et al., 2022). Indeed, over 90% of the DNA isolated from breast milk is of human instead of microbial origin (Jiménez et al., 2015). Host depletion methods utilizing benzonase or PMA have proven inefficient in enriching microbial DNA (Spreckels et al., 2023). Additionally, these methods are also demanding as they necessitate fresh samples and immediate processing (Liu et al., 2023). Therefore, most studies of milk microbiota have relied on 16S rRNA amplicon sequencing, with the limitation of low taxonomic resolution and an inability to study microbial transmission (Pannaraj et al., 2017; Wang et al., 2020). Metagenomic sequencing could overcome these limitations, but it is methodologically challenging due to the low percentage of microbial reads in the samples. To date, very few studies have successfully employed shotgun metagenomics sequencing for human milk microbiota, with most reporting very low microbial reads after filtering (Asnicar et al., 2017).

Microbiome analysis on infant fecal samples, especially meconium, however, faces the issue of low DNA amounts rather than high host contamination. Newborns, just a few days old, are characterized by possessing low quantities and diversity of gut microbiota (Wang et al., 2020). Current studies even disagree on whether detectable meconium microbiota exist (Perez-Muñoz et al., 2017; Kennedy et al., 2021; Stinson et al., 2019). Low DNA input may lead to inaccurate 16S results and interfere with the library preparation step of metagenomic sequencing (Westaway et al., 2021). Therefore, it is crucial to establish an approach to overcome both host contamination and low biomass problems for a more in-depth study on early microbial colonization with larger sample sizes and varied collection sites.

Here, we performed comparative microbiome analyses on human breast milk and infant first-pass fecal samples by employing Type IIB Restriction Enzymes Site-Associated DNA sequencing for microbiome (2bRAD-M) with 16S rRNA amplicon and metagenomic sequencing. 2bRAD-M specifically targets and unbiasedly amplifies DNA fragments containing specific sequences generated by Type IIB restriction enzymes (Sun et al., 2022). Then the reads will be mapped to pre-built 2bRAD tag database to identify species and estimate their abundances. Since 2bRAD utilizes multiple species-specific markers to jointly confirm the presence of microbes in the community, it could achieve higher accuracy than 16S in microbial profiling. In comparison to WMS, 2bRAD method was known as a reduced metagenomic way to capture microbial signals with only 1% genetic content per genome. As such, microbial identification requires far less sequencing depth per genome, then it is more capable in identifying low-abundance microbes in the communities, providing complete microbial landscapes. Although studies have demonstrated that this method is capable of accurately profiling the species-level microbial composition of samples of mock communities (Sun et al., 2022), oral (He et al., 2023), fecal (Zhang et al., 2023), urine (Hong et al., 2022), intratumoral (Xue et al., 2024), and environmental (Lam et al., 2023) microbiota, its applications in extremely low abundant maternal and infant microbiomes remain unexplored. Thus, the present study investigated the robustness and validity of 2bRAD-M on human milk and infant meconium samples and explored the translational potential.

## Materials and methods

### Human subjects

A total of 41 Chinese healthy pregnant women (30.1 ± 2.5 years old) in their third trimester (34–36 gestational weeks) were recruited from our present cohort jointly by Divisions of Obstetrics and Stomatology, Shenzhen Maternal and Child Healthcare Hospital (SMCHH) (Zhao et al., 2024). Prior to the study, all participants provided informed written consent. The study protocol was approved by the SMCHH Ethics Committee (SFYLS[2020] 013) and conducted following the current Declaration of Helsinki. Samples of breast colostrum/milk and feces from mothers and meconium/feces from their infants were collected during the perinatal period and at 6 months postpartum check-up. The first-form meconium sample was collected while the medical examination was performed on newborns. At 6 months postpartum, additional maternal breast milk samples were collected. The medical and biochemical datasets were retrieved from the internal records of the cohort of SMCHH. Questionnaire information covered dietary habits, smoking, and drinking habits, menstrual and reproductive history, previous adverse pregnancy outcomes, history of medication use (especially hormonal medications), BMI, history of exposure to radiation and harmful substances, history of immune system diseases, and genetic history.

Inclusion criteria included non-smoking, non-drinking women with regular dietary habits (excluding vegetarians or those on a diet), a normal pre-pregnancy body mass index (BMI), overall good health without systemic diseases (as outlined in the exclusion criteria), no antibiotic use within at least the past 3 months.

The subjects with the following conditions were excluded from the study: (i) not singleton pregnancy, (ii) systemic diseases such as diabetes, cardiovascular diseases, and cancer; (iii) infectious diseases such as HIV, hepatitis B, and syphilis; (iv) the ongoing use of immunosuppressants, bisphosphonates for osteoporosis, or steroid and immune-related drugs, (v), history of recurrent preterm births, previous adverse pregnancy outcomes, and requiring assisted reproductive technologies; (vi) immune system diseases; (vii) age over 35 years; (viii) pre-pregnant BMI ≥ 28 kg/m^2^.

### Sample collection and DNA extraction

Subjects were instructed to collect fresh fecal samples using a sterile feces tube, and the screw cap (Sarstedt) was provided by the research team. Samples were immediately transported to the laboratory in a cold pack. Approximately 1 g of maternal fecal sample was collected from the internal center of freshly obtained feces, and another 20 grams were stored at −80°C. Meconium samples were collected within 12–24 h postpartum, right at the time of first pass, using a disposable diaper. After collection, the samples were immediately placed into sample bags, processed under sterile conditions, and stored at −80°*C. prior* to colostrum or milk sample collection, both sides of the participant’s nipples, areolae, and surrounding skin were disinfected using a chlorhexidine wipe to reduce contamination by skin microbiota. The first drop of breast milk (approximately 500 μL) was discarded, after which the sample was collected and stored at −80°C. Breast milk samples were defatted by ultracentrifugation before DNA extraction. DNA from 200 μL maternal milk samples was extracted by TIANGEN’s TIANamp Micro DNA Kit (Cat. No. DP316, Supplementary material). DNA from 200 mg mother fecal and infant meconium samples was extracted by cetyltrimethylammonium bromide (CTAB) method (Supplementary material).

### The 16S amplicon sequencing and data analysis

The V3–V4 regions of the 16S rRNA were amplified with barcoded primers 341F (5′-CCTAYGGGRBGCASCAG-3′) and 806R (5′-GGACTACNNGGGTATCTAAT-3′) for library preparation. To ensure the absence of contamination, two blank controls were incorporated for each sample during the amplification process. Sequencing was carried out on the Illumina NovaSeq 6000 Systems (Illumina) utilizing paired-end sequencing (PE250) with 50,000 tags per sample (Novogene, Beijing, China). Rawdata was quality controlled by FastQC^1^ (Babraham, 2024) (Supplementary Table S1). Then, they are imported into QIIME2 (v.2023.2) (Bolyen et al., 2019) for subsequent analysis. Reads were denoised using DADA2 (Callahan et al., 2016) (denoise-paired, v.1.26.0), and feature tables with amplicon sequence variants (ASV) profiles were generated. Taxonomies were assigned to ASVs using a pre-trained classifier (Genome Taxonomy Database (GTDB) Full R202 database) (Parks et al., 2022). Feature tables of relative abundances at both genus and species levels were exported. Subsequent analyses were carried out using R (v.4.2.2). Of 30 meconium samples, we have an average read of 76 k after quality check. Among them, two of the samples had higher than 90% reads unassigned at the genus level, which were discarded in downstream analysis.

### 2bRAD-M sequencing and data processing

All specimens were sequenced using a reduced metagenomics approach, specifically 2bRAD-M (Sun et al., 2022), at Qingdao OE Biotech Co., Ltd. (Qingdao, China). Blank controls were included to remove contamination during the experimental pipeline. Genomic DNA was digested with 4 U of the Type IIB restriction enzyme (BcgI) for 3 h at 37°C. The digestion reaction was followed by ligation in a 10 μL mixture containing the digested DNA, T4 DNA Ligase Buffer, 1 mM ATP (NEB), 0.2 μM of adaptors (Ada1 and Ada2), and 800 U of T4 DNA ligase (NEB). This ligation reaction lasted 16 h and was inactivated by heating at 65°C for 20 min. For DNA amplification, PCR was performed in a 40 μL reaction volume, which included 7 μL of ligated DNA, 1× Phusion HF buffer, 0.1 μM of Illumina primers, 0.3 mM dNTP, and 0.4 U of Phusion high-fidelity DNA polymerase (NEB). The PCR cycling conditions were 16 to 28 cycles with an initial denaturation at 98°C for 5 s, annealing at 60°C for 20 s, extension at 72°C for 10 s, and a final extension at 72°C for 10 min. The target fragments, approximately 100 bp in size, were excised from an 8% (wt/vol) polyacrylamide gel and extracted in nuclease-free water at 4°C for 12 h. Sample-specific barcodes were then added through another PCR step using primers with platform-specific barcodes. The 40 μL PCR reaction contained 50 ng of the purified product, 0.2 μM of each primer, 0.6 mM dNTP, 1× Phusion HF buffer, and 0.8 U of Phusion high-fidelity DNA polymerase (NEB). Finally, the PCR products were purified using the QIAquick PCR purification kit (Qiagen, Valencia, CA) and sequenced on the Illumina Novaseq 6000 platform.

The initial step involved filtering raw reads using the FastQC (See text footnote 1) (Babraham, 2024) and subsequently processing them to isolate the specific digested fragments identified by the BcgI recognition site. Clean reads were generated by removing reads with more than 8% unknown bases and discarding low-quality reads in which over 20% of the bases had a quality score below Q30. Quality control report for the pre-processing was included (Supplementary Table S2). Of all 97 samples subjected to 2bRAD-M sequencing, they generated an average of 13 M raw reads and 10 M clean reads after the quality control. Only one meconium sample has a <10% percentage of reads passing quality check, which may be due to sample degradation. This sample was discarded for downstream analysis. Utilizing 2bRAD-M sequencing data consisting of 32-bp iso-length reads, our computational pipeline, 2bRAD-M,^2^ was employed to characterize species-level microbial communities. This bioinformatics pipeline depends on a pre-existing 2bRAD tag database (2bTagDB), containing species-specific markers derived from a comprehensive dataset of 258,406 microbial genomes obtained from GTDB. Microbial species were identified for each microbiota using the GTDB, and their relative abundance was estimated by normalizing the sequencing coverage of single-copy, species-specific markers (Sun et al., 2022). Specifically, the average read coverage of 2bRAD markers for each species was calculated to reflect the number of individuals from that species in the sample. The relative abundance was then determined by dividing this coverage by the total number of individuals from all identified species within the sample.

To identify and remove potentially contaminant species, FEAST (Shenhav et al., 2019) (v.1.0.2), decontam (Davis et al., 2018) (v.1.10.0), and microDecon (McKnight et al., 2019) (v.0.1.0) were employed to perform the post-sequencing decontamination using negative controls sequenced by 2bRAD-M. For each species which was identified by decontam or microDecon as a contaminant species, if its FEAST-calculated probability originating from each “Source” (negative control) was greater than zero, this species was removed according to the probability ratio. Species not identified as contaminant species by decontam or microDecon were retained.

### Metagenomics sequencing and data analysis

The genomic DNA was also subject to shotgun metagenomics sequencing using DNBSEQ-T7 platform (BGI, Shenzhen, China) (Kim et al., 2021). The metagenomic raw data were quality-controlled using Kneaddata (v.0.12.0, huttenhower.sph.harvard.edu/kneaddata/) with options --trimmomatic $my_path -t 30 --bypass-trf. FastQC (See text footnote 1) (Babraham, 2024) was used for generating QC report (Supplementary Table S3). For taxonomic profiling of metagenomic samples and comparing the profiling performance of WMS with 2bRAD-M, it is essential to use a consistent database (i.e., GTDB) for all sequencing data types. Therefore, those taxonomic profilers that do not support for building a custom database, such as MetaPhlAn and mOTU, were not used. Since both 16S sequencing and 2bRAD-M profile taxonomic abundance, sequence abundance profilers were not suitable in our settings (Sun et al., 2021). To maximize the impact of database composition on our benchmarking work, we chose to digitally digest WMS sequencing data and used 2bRAD-M pipeline to profile their relative abundance using GTDB.

### Statistical analysis and visualization

The statistical analysis was conducted with R (v.4.2.2). The profiling results of all three sequencing methods were imported to R in the form of feature tables. The Alpha diversity and Beta diversity analyses were performed in R using phyloseq (McMurdie and Holmes, 2013) (RRID: SCR_013080; v.1.42.0). The feature table was first used for Alpha diversity estimation, including Shannon index, etc. For Beta diversity estimation, UniFrac distance was calculated for each pair of samples derived from each sequencing method, based on the fraction of branch length shared between two communities within the GTDB bac_202 tree (Parks et al., 2018).

A total of 130 clinical variables were collected from each participant. We calculated the percentage of missing data for each variable, and variables with more than 20% missing data were discarded from the analysis. To identify the microbial differences among groups defined by different metadata variables (Supplementary Table S4), we converted continuous variables to deciles. Then we performed PERMANOVA test based on species-level beta diversity (weighted UniFrac distance). PERMANOVA was performed in R using the adonis2 function in vegan (Oksanen et al., 2024) (RRID: SCR_011950; v.2.6-4). A threshold (*p*-value < 0.05) was set to identify clinically significant metadata variables in all three sample types. Principal Coordinate Analysis (PCoA) based on a given distance metrics was conducted to reduce the dimensionality of microbiome data for better visualization. The resulting first two principal coordinates (i.e., PC1 and PC2) were visualized via a scatter plot using the R package ggplot2 (Wickham, 2009) (RRID: SCR_014601; v.3.3.3). Low-abundance (relative abundance <0.1%) species were filtered using R package metagMisc (v.0.5.0) and differential abundance analysis was carried out by Linear Discriminant Analysis Effect Size (LEfSe) (Segata et al., 2011).

## Results

### 2bRAD-M enables successful sequencing of low-biomass meconium and breast milk samples compared to WMS and 16S sequencing methods

**Figure 1:**
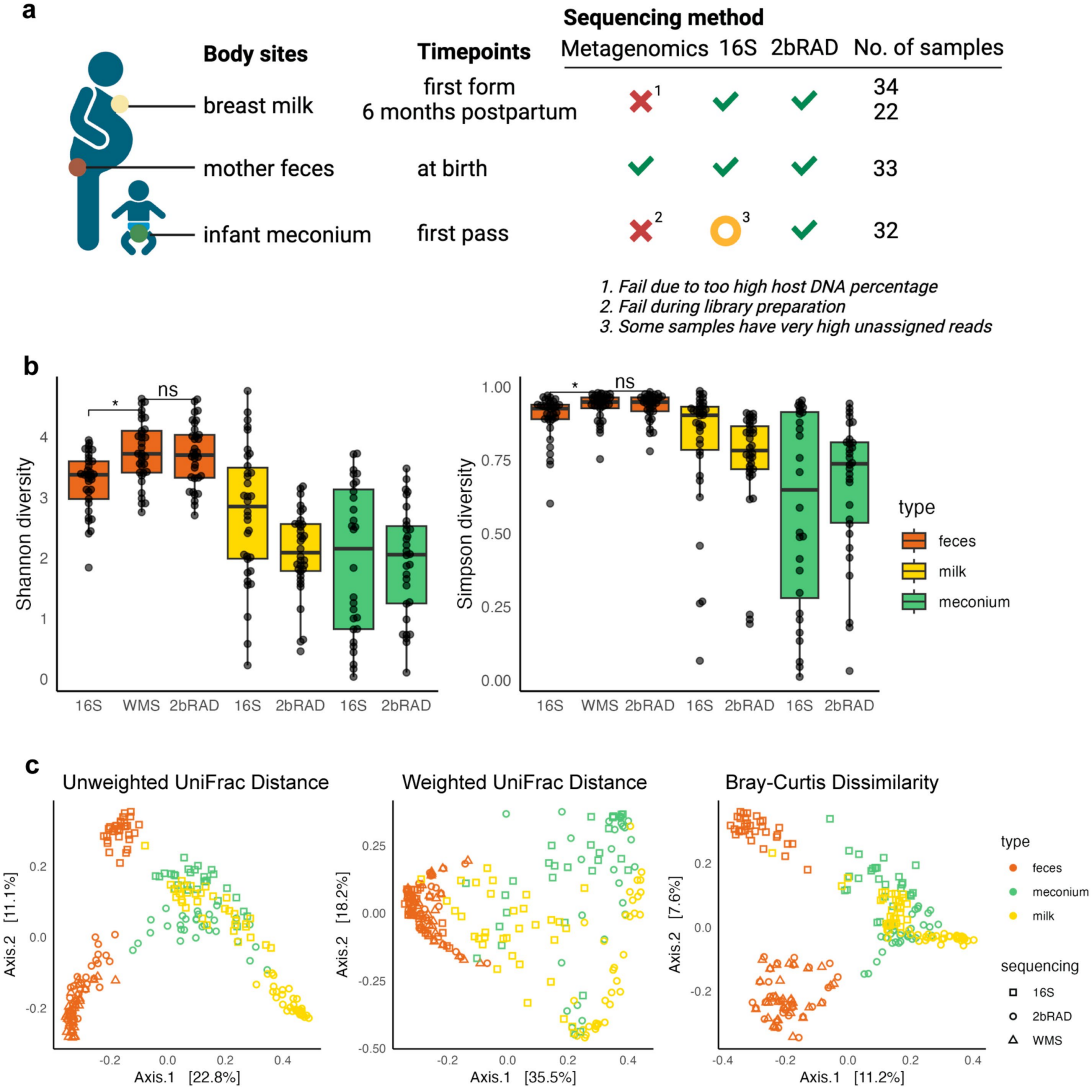
2bRAD-M is a robust sequencing method compared to WMS and 16S. **(A)** Study design and sequencing overview. Samples of breast milk, mother feces and infant meconium were sequenced. Breast colostrum/milk samples were collected at two time points: first form and 6 months postpartum. Maternal feces were collected during the perinatal period, and meconium samples were collected at their first pass. Whole metagenomics sequencing (WMS), 16S rRNA amplicon sequencing, and 2bRAD-M were carried out for each sample. Green ticks mean successful sequencing for all samples. Orange circles show problems for some of the samples. Red cross stand for failure for all samples. **(B)** Alpha diversities of mother fecal samples using three different sequencing methods is shown. The different sample types are color-coded: feces (red), milk (yellow), and meconium (green). Shannon diversity index (left) and Simpson diversity index (right) varied between 16S and gold standard WMS (Wilcoxon rank sum test, adjust *p* = 0.010 and 0.034), but not between 2bRAD-M and WMS (Wilcoxon rank sum test, adjust *p* = 0.809 and 0.970). **(C)** PCoA plot for all samples with different sequencing methods based on unweighted UniFrac distance, weighted UniFrac distance, and Bray–Curtis dissimilarity between microbial communities (*N* = 225). Panel **(A)** was created in BioRender (Ref No.: BioRender.com/k60f988).

In our cohort study (Zhao et al., 2024), we recruited 41 pregnant women in the third trimester ready for delivery to collect breast milk, feces, and infant meconium samples (Figure 1A). The clinical characteristics of the study participants were summarized (Supplementary Table S5), including maternal age, pre-pregnancy BMI, fetal sex, mode of delivery, and fetal birth weight.

First-form breast milk samples were obtained from 34 women, with follow-up samples collected from 22 of these women after 6 months. The six-month follow-up was incomplete due to the pandemic. Maternal fecal samples were collected from 33 mothers. The first-pass feces (meconium) samples were collected from 30 infants. All samples were subjected to WMS, 16S rRNA amplicon sequencing (16S), and 2bRAD-M sequencing.

Notably, 2bRAD-M yielded high-quality data with no failures in library preparation. After quality checks, all samples obtained at least 4 million paired-end reads (Supplementary Figure S1; Supplementary Table S6). In contrast, WMS failed for both milk and meconium samples and only succeeded in high-biomass fecal samples. Specifically, all 30 infant meconium samples failed in library preparation due to low DNA input. For the 34 human breast milk samples, WMS produced an average of over 100 million reads per sample, but only three samples yielded over 0.5 million microbial reads after filtering out human-derived reads (Supplementary Table S7), rendering WMS unsuitable for milk samples. All 34 maternal fecal samples provided over 200 million total reads (260 million reads/sample on average) for WMS sequencing and were used as the gold standard for subsequent inter-method paired analysis. While 16S sequencing was technically successful for all samples, some exhibited a high proportion (over 90%) of unassigned reads, highlighting its limited compatibility of with low biomass samples.

### 2bRAD-M captures the microbial ecology difference among the mother’s feces, breast milk, and meconium

To compare the microbial composition results obtained from different sequencing methods and sample types, we performed taxonomic profiling of all samples using the GTDB reference database (Supplementary Table S8). We observed higher alpha diversity indices in fecal samples when using WMS compared to the 16S (*p* < 0.05), but not to 2bRAD-M (Figure 1B), implying that the 2bRAD-M provides diversity estimates comparable to those of the gold standard WMS. Comparing different sample types, we can observe a higher species richness and evenness in mother fecal samples than in mother breast milk and infant meconium samples.

We next clustered species-level profiles of infant meconium, maternal breast milk and fecal samples using PCoA based on the UniFrac distance and Bray–Curtis dissimilarity, respectively (Figure 1C). Infant meconium samples and maternal breast milk samples clustered closely together, while mother fecal samples were separated from them. This indicates that the microbial communities of infant meconium and mother breast milk are more similar to each other compared to mother fecal samples, indicating potential mother-to-infant transfer of microbiota through lactation. Furthermore, for the maternal fecal samples sequenced by three different methods, we found that those sequenced using 16S clustered separately from those sequenced by the gold standard WMS and 2bRAD-M, echoing a closer resemblance in performance of the two methods.

### 2bRAD-M demonstrates a robust and accurate sequencing method based on maternal fecal samples

**Figure 2:**
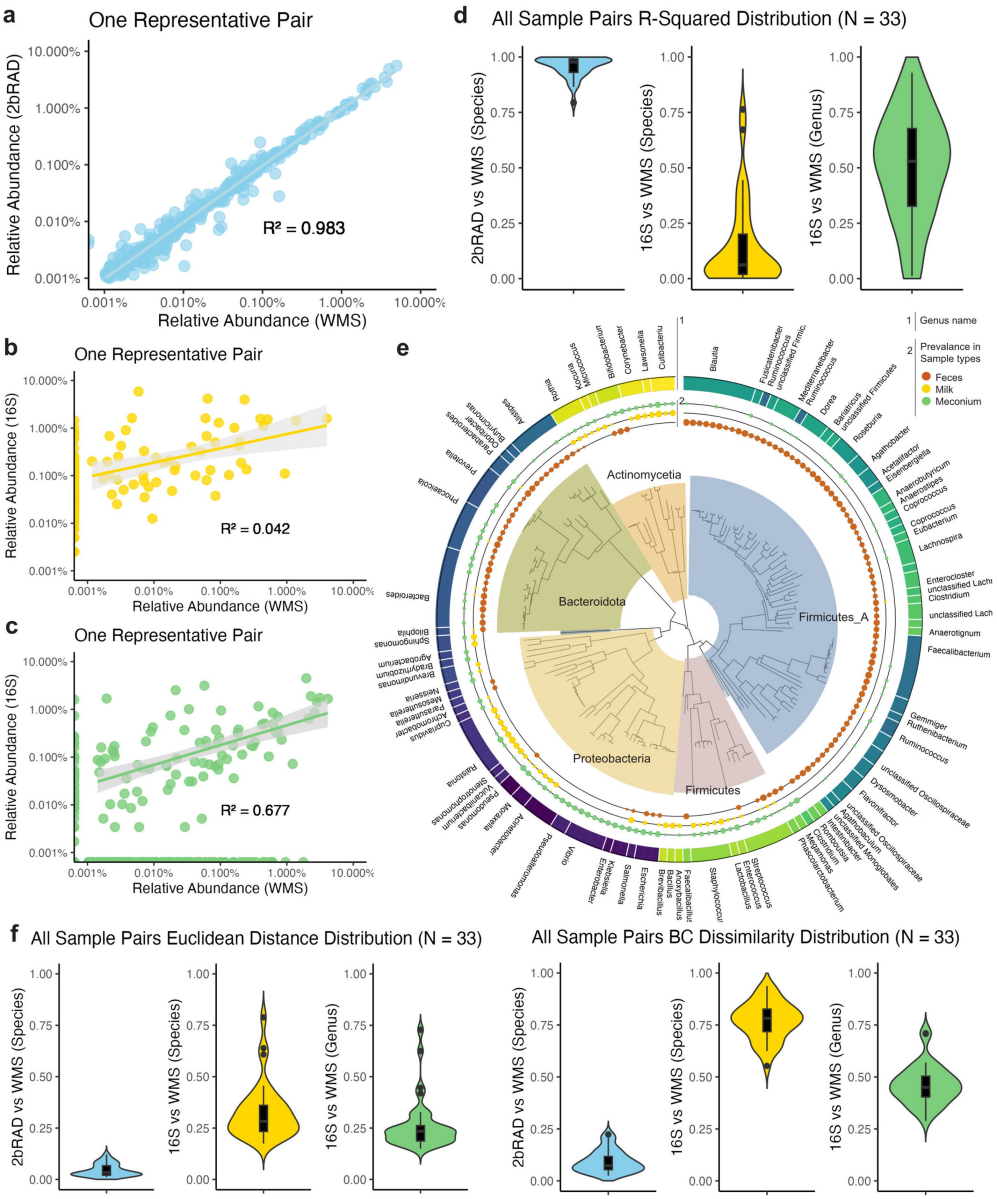
Pairwise analysis of correlation and distance between sequencing methods in maternal fecal samples. **(A)** Scatter plot of one representative sample comparing the species-level relative abundance of 2bRAD-M to that of the gold-standard WMS method. Each point represents the relative abundance of a detected species within one sample. Linear regression models are fitted to the data, shown by the solid line, with the 95% confidence interval indicated by the shaded area (*R*^2^ = 0.983, *p* = 0.002). **(B)** Scatter plot of one representative sample comparing the species-level relative abundance of 16S to that of the WMS (*R*^2^ = 0.444, *p* = 0.002). **(C)** Scatter plot of one representative sample comparing the genus-level relative abundance of 16S to that of the WMS (*R*^2^ = 0. 677, *p* = 0.013). **(D)** R squared of linear regression of all 33 sample pairs compared with the gold standard WMS are shown in the violin plots. For 2bRAD-M, it had a high and robust correlation (*M* = 0.97, *SD* = 0.05). For 16S, both species-level and genus-level correlations are low and had a large variance (*M* = 0.06, *SD* = 0.20 and *M* = 0.53, *SD* = 0.24). **(E)** Euclidean distance and Bray–Curtis dissimilarity between all 33 sample pairs are shown in the violin plots. Microbiome composition measured by 2bRAD-M and WMS at species-level exhibits very high similarity (L2 *M* = 0.03, *SD* = 0.03 and BCD *M* = 0.07, *SD* = 0.05), while that of 16S and WMS at both species (L2 *M* = 0.28, *SD* = 0.14 and BCD *M* = 0.78, *SD* = 0.09) and genus-level (L2 *M* = 0.23, *SD* = 0.12 and BCD *M* = 0.45, *SD* = 0.09) showed high variability. **(F)** Phylogenetic representation of the prevalence of keystone species (filtered by abundance and overall prevalence) in three sample types: feces, milk, and meconium. The size of the points is proportional to the prevalence of the species in the respective sample types. The genus information is shown as colored with names annotated. The tree is annotated with phylum-level information using different colors.

To further demonstrate the performance (robustness and accuracy) of 2bRAD-M sequencing, we conducted pairwise comparisons of the taxonomic profiling results from three sequencing methods. We compared the outcomes of both 2bRAD-M and 16S against WMS results as the gold standard. Noteworthily, 2bRAD-M exhibits a strong correlation with WMS in terms of species-level relative abundance within one sample (*R*^2^ = 0.983, *p* = 0.001, Figure 2A). In contrast, the 16S rRNA gene sequencing method displayed a markedly weaker correlation with WMS. The discrepancy is evident at both the species-level (Figure 2B) and genus-level (Figure 2C) relative abundances, highlighting the limited reliability of 16S sequencing for precise taxonomic profiling. The detailed analysis of all sample pairs (*N* = 33) further supported our findings (Supplementary Figures S2–S4). The regression analyses showed the superior performance of 2bRAD-M, with consistently high R-squared values (*M* = 0.97, *SD* = 0.05) across all pairs. Conversely, the linear regression models for the 16S data exhibited large variance and significantly lower R-squared values (*M* = 0.06 for species and 0.53 for genus, Figure 2D).

In addition to correlation analysis, we also quantified the cross-method Euclidean distances (L2) and Bray-Curtis dissimilarities (BCD) across all 33 sample pairs (Supplementary Table S9). For species-level profiles, we observed a low 2bRAD-M-to-WMS distance (Euclidean distance and BCD), suggesting a high cross-method similarity in the species-level composition of the same samples (L2 *M* = 0.03, *SD* = 0.03 and BCD *M* = 0.07, *SD* = 0.05; Figure 2E). Conversely, 16S exhibited significant low technical stability in the genus or species-level profiling when compared to WMS. The cross-method differences (i.e., Euclidean distances and BCD) in taxonomic profiles are notably high at either genus (L2 *M* = 0.28, *SD* = 0.14 and BCD *M* = 0.78, *SD* = 0.09) or species level (L2 *M* = 0.23, *SD* = 0.12 and BCD *M* = 0.45, *SD* = 0.09). This low technical stability shows the inadequacy of 16S in capturing the diversity and microbial composition.

We further presented the phylogenetic distribution of the prevalence of each species for the three sample types: maternal breast milk, feces, and infant meconium (Figure 2F). Our findings revealed a niche-specific distribution of microbial communities across different sites. Specifically, we observed that the phylum *Firmicutes_A* was enriched in feces, whereas the phylum *Firmicutes* were more prevalent in milk and meconium. In the phylum *Proteobacteria*, the orders *Pseudomonadales* and *Enterobacterales* were enriched in infant meconium, while *Burkholderiales* were more frequently found in milk samples. These results aligned with previous studies indicating similar microbial compositions in these environments. It has been reported that fecal samples predominantly contain *Firmicutes*, *Proteobacteria*, and *Bacteroidetes*. Milk mainly has *Firmicutes* and *Proteobacteria*. Meconium is mostly composed of *Firmicutes* and *Actinomycetes* (Banić et al., 2022).

### 2bRAD-M enables associating meconium microbiota with a wide range of clinical covariates

**Figure 3:**
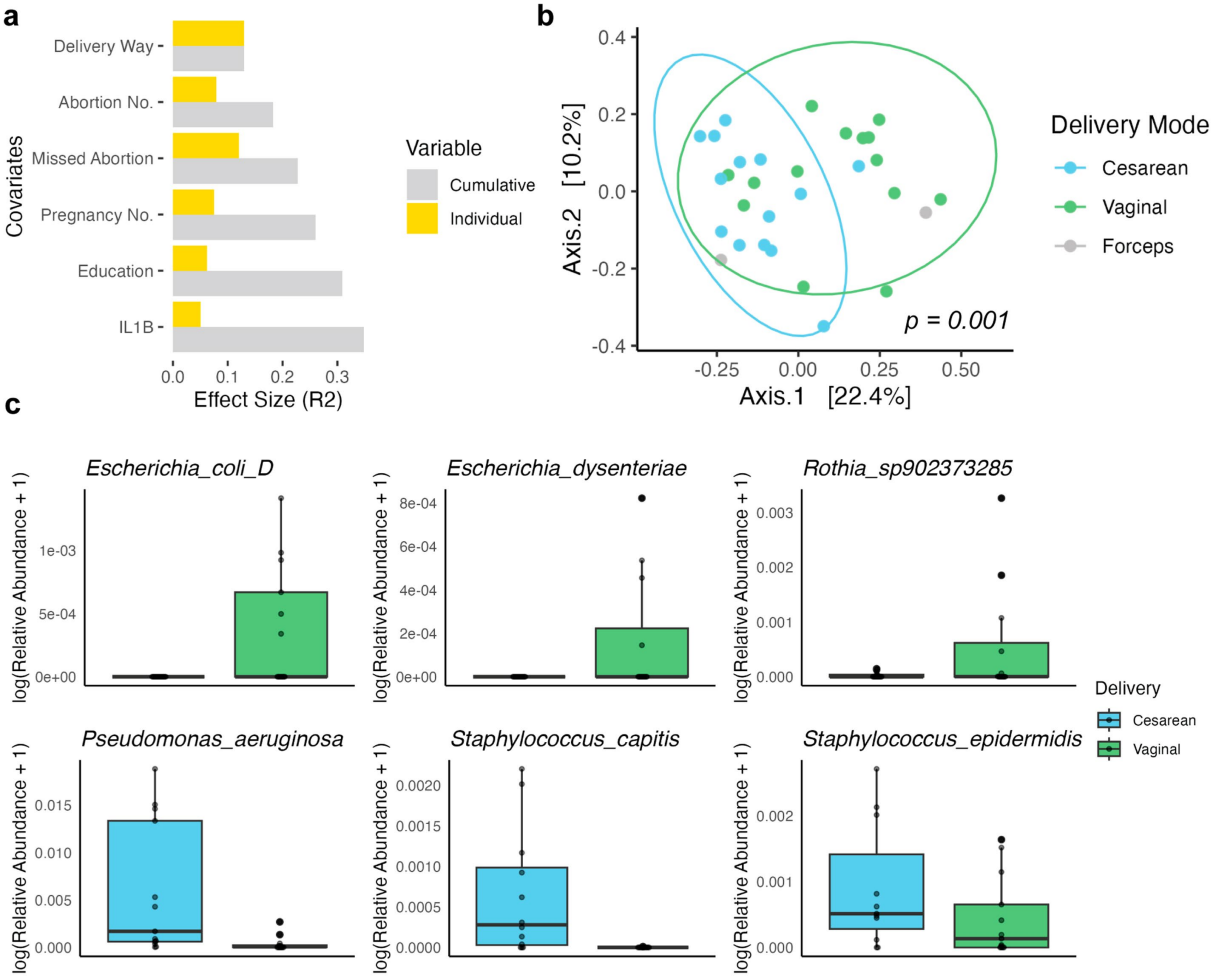
Clinical metadata variables associated with microbial beta diversity in infant meconium samples. **(A)** Cumulative effect size (R^2^) of significant covariates (sorted) on microbiota community variation (weighted UniFrac distance) as compared to individual effect sizes. **(B)** PCoA on weighted UniFrac distance representing species-level microbiota variation for infant meconium samples. PCoA1 (Axis.1) and PCoA2 (Axis.2) explained 22.4 and 10.2%, respectively, of the variance. Each dot represents one sample, colored by the delivery mode. **(C)** Boxplots displaying the relative abundance (log-transformed) of keystone bacterial species associated with different delivery modes: cesarean (blue) and vaginal (green).

We next explored the underlying associations between 2bRAD-M-resolved microbiota and a wide range of clinical covariates. No significant clinical variable was found in the maternal fecal samples. Interestingly, notable differences were observed in the breast milk microbiota profiles between participants who consumed grains and beans “sometimes” versus “every day” (PERMANOVA, *p* = 0.045, *F* = 2.11). In the analysis of infant meconium samples, we identified six covariates (delivery way, abortion number, missed abortion, pregnancy number, education level, and Interleukin-1 beta (IL1B) level, Supplementary Table S10) associated with microbial variation, with the delivery way having the highest explaining value of 13% (Figure 3A), in line with previous studies (Banić et al., 2022; Bäckhed et al., 2015). We also observed additional factors, including the mother’s history of abortion and number of pregnancies, also contributed to the infant’s microbial profile, with these six covariates collectively explaining 34.8% of the compositional variance (Supplementary Table S11).

A significant separation between delivery modes in infant meconium microbiome was revealed in PCoA (Figure 3B). We next identified differentially abundant species in the meconium between delivery modes using LEfSe (Wilcoxon test, adjust *p* < 0.05, LDA score > 3, Supplementary Table S12), and presented the keystone species with most differential abundance (Figure 3C). Bacterial species typically present in the maternal gut such as *Escherichia coli*, *Escherichia dysenteriae,* and a *Rothia* species were found in higher abundance in vaginally delivered infants. In contrast, bacterial species more commonly associated with the environment or maternal skin like *Pseudomonas aeruginosa*, *Staphylococcus capitis,* and *Staphylococcus epidermidis* exhibited higher relative abundance in C-section infants.

### 2bRAD-M enables the high-resolution longitudinal study of breast milk microbiome

**Figure 4:**
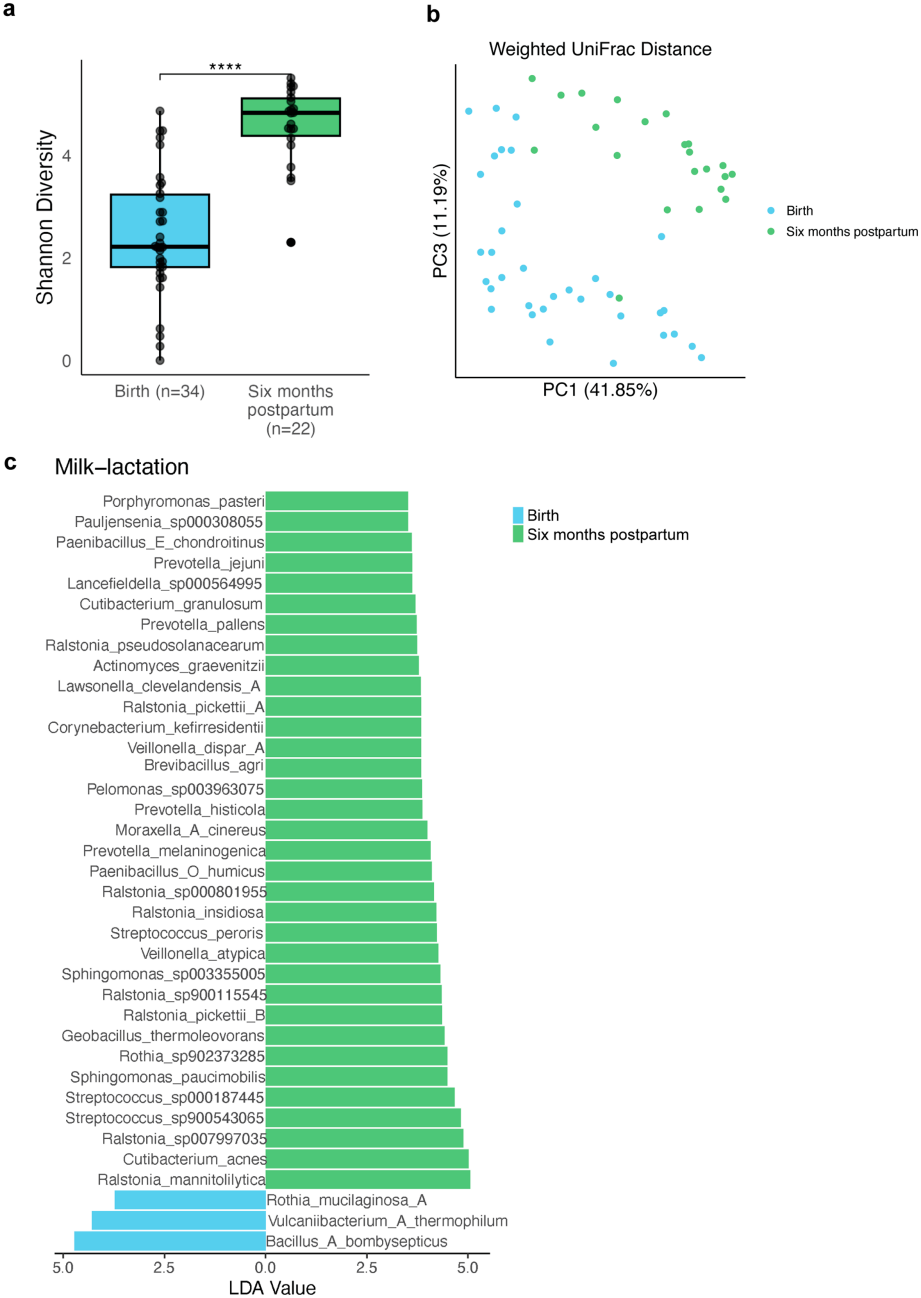
Human breast milk microbiome diversity changes through different lactation stages. **(A)** Shannon diversity of breast milk samples collected at the time of the child’s birth (green) and 6 months postpartum (blue). (Wilcoxon rank sum test, adjust *p* < 0.001). **(B)** PCoA plot for breast milk samples taken at different time points based on species-level weighted UniFrac distance between microbial communities. **(C)** Linear discriminant analysis (LDA) plot identifies the differential abundance of microbes in human milk during lactation. Blue indicates the birth group. Green indicates the six-month postpartum group. Only taxa with LDA score greater than 3.5 are shown.

Due to the high host contamination, the maternal breast milk samples in previous research could only be successfully sequenced using 16S sequencing which has limited resolution. Utilizing 2bRAD-M to successfully reach high-resolution sequencing of milk microbiota, we further collect milk samples from 22 mothers at 6 months postpartum to explore the microbial diversity along lactation stages. Previous studies reported the increase of milk microbial diversity through the lactation stage at the genus level (Singh et al., 2023). Through 2bRAD-M, we observed a significantly higher species-level Shannon diversity in samples collected at 6 months postpartum (Figure 4A), indicative of increased microbial diversity possibly due to microbial transmission during lactation. Breast-milk microbiotas from the initial and later stages of lactation were clearly segregated in the PCoA plot based on species-level weighted UniFrac distance (Figure 4B; PERMANOVA, *p* = 0.001). Next, we employed LEfSe to identify the key distinguishing lactation-stage-associated microbial species in breast milk. Though the relative abundance of the dominant genera *Streptococcus* was reported to be stable during lactation (Wan et al., 2020), our data indicated the enrichment of three *Streptococcus* species 6 months postpartum. Additionally, *Veillonella atypica* and *Veillonella dispar* were found to be enriched 6 months postpartum, contributing to the transfer of facultative anaerobes to the infant gut (Figure 4C).

## Discussion

While there have been extensive studies on maternal and infant microbiome, the challenge of analyzing low biomass samples, especially breast milk, has not been well addressed (Ferretti et al., 2018; Wan et al., 2020; Fang et al., 2024). This study employed innovative 2bRAD-M sequencing technology (Sun et al., 2022) to profile the microbial communities of infant meconium, maternal fecal and breast milk samples and performed benchmarking with traditional metagenomics and 16S sequencing. The 2bRAD-M approach demonstrated a high correlation with whole metagenome sequencing (WMS), consistently outperforming the traditional 16S rRNA sequencing. This high level of accuracy, resolution, and ability to process low-biomass samples stems from the use of reduced metagenomics that employs uniform-length fragments covering the entire genome. To our knowledge, it is the first time that the species-level microbial composition of breast milk has been successfully profiled. This is a great step forward to raise the resolution of milk microbiota analysis.

In alignment with previous findings indicating that the mode of delivery is a significant factor influencing an infant’s gut microbiome (Mitchell et al., 2020; Reyman et al., 2019), we further identified the impact of various clinical variables on infant meconium. Delivery way, abortion number, missed abortion, pregnancy number, education level, and IL1B level were all found to be factors leading to microbial variations. Among them, delivery mode significantly shaped microbial profiles, with two *Escherichia* species predominating in vaginally delivered infants and some environmental microbiome species being more abundant in cesarean-delivered infants. Moreover, previous studies have found that maternal diet influences human milk oligosaccharides composition and milk microbiota (Seferovic et al., 2020; Taylor et al., 2023). In line with this, we observed notable differences in breast milk microbiota profiles between mothers with different consumptions of grains and beans, supporting diet as a factor shaping the milk microbiome.

Consistent with previous findings indicating the increase in genus-level milk microbial diversity across lactation stages using 16S sequencing (Singh et al., 2023), we derived similar conclusions but at species-level using 2bRAD-M sequencing. Moreover, previous studies have shown genus *Veillonella* enriched in mature milk along lactation stage (Singh et al., 2023). Consistently, we observed an enrichment of three *Streptococcus* species and two *Veillonella* species 6 months postpartum. These facultative anaerobes are known to play a critical role in early gut colonization, potentially influencing infant immune system development (Ferretti et al., 2018). *Veillonella* is commonly associated with lactic acid utilization in the infant gut, facilitating the metabolism of maternal breast milk (Bäckhed et al., 2015). This longitudinal increase may indicate microbial adaptation to evolving nutrient availability in breast milk, such as changing concentrations of human milk oligosaccharides (Pannaraj et al., 2017). These findings offer insights into potential applications such as the modification of maternal diet and targeted probiotics to support the establishment of a healthy infant microbiome.

One notable limitation of our study is the relatively small size of our cohort. This restricts the ability to conduct mother-infant vertical transmission analysis and explore possible associations between microbial biomarkers and infants’ health status. Despite this, the demonstrated accuracy and robustness of 2bRAD-M underscore its potential as a transformative tool in microbiome research. Future research that involves larger cohorts with longitudinal sampling can significantly enhance our understanding of microbial transmission dynamics between mothers and infants, uncovering the mechanistic understanding of factors shaping infant microbiomes. Such insights are essential for personalized interventions (e.g., infant microbiome seeding) aimed at optimizing the early-life microbial colonization for improved health outcomes.

The utility of 2bRAD-M extends far beyond maternal and infant microbiome studies. Its ability to deliver high-resolution microbial profiles from low-biomass samples positions it as a valuable tool for resolving longstanding challenges in other clinical contexts. For example, it could help definitively address the global debate on the presence of microbes in amniotic fluid (Stinson et al., 2019; Lim et al., 2018), where conventional methods have fallen short due to technical limitations. Additionally, the method’s versatility makes it well-suited for profiling other challenging clinical samples, such as blood (Qian et al., 2023) and tumor samples (Battaglia et al., 2024), where 16S amplicon sequencing features low-resolution, and off-target issues and WMS suffers from the ultra-high sequencing cost to detect rare microbes from host-rich samples.

The demonstrated potential for 2bRAD-M to identify species-level microbial biomarkers opens new possibilities for clinical applications, including the development of DNA-based diagnostic tools. By designing the DNA probes from microbial biomarkers detected by 2bRAD-M, we can develop novel methods for rapid and precise detection of microbial signatures linked to chronic diseases. In conclusion, our study not only marks a step forward in understanding the maternal and infant microbiome but also set the stage for broader applications in clinical microbiome research, paving the way for innovative diagnostic strategies and personalized medicine.

## Data Availability

The datasets presented in this study can be found in online repositories. The names of the repository/repositories and accession number(s) can be found below: https://ngdc.cncb.ac.cn/search/specific?db=bioproject&q=PRJCA030517.

## Footnotes

^1^http://www.bioinformatics.babraham.ac.uk/projects/fastqc/, v0.11.9.

^2^ https://github.com/shihuang047/2bRAD-M
