# Supplementary material for: Unveiling early-life microbial colonization profile through characterizing low-biomass maternal-infant microbiomes by 2bRAD-M
Source: Front Microbiol. 2025 Jan 24;16:1521108. doi: 10.3389/fmicb.2025.1521108 (PMC11802558; doi:10.3389/fmicb.2025.1521108)
Supplement: Supplementary file 1 [file Data_Sheet_1.pdf]

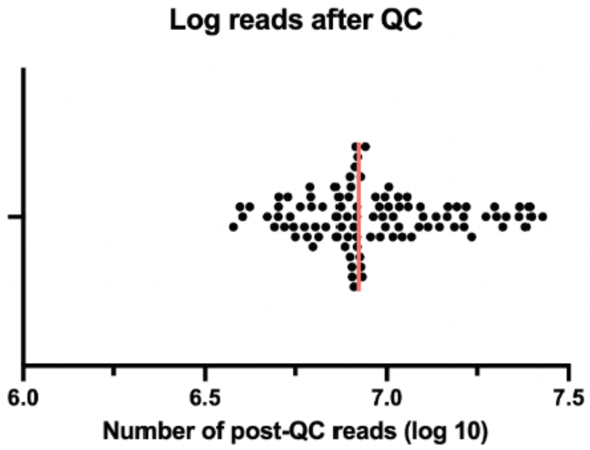

Fig. S1 Boxplots of the sequencing reads of all samples sequenced by 2bRAD-M (N = 97) after quality control. The red line indicates median.

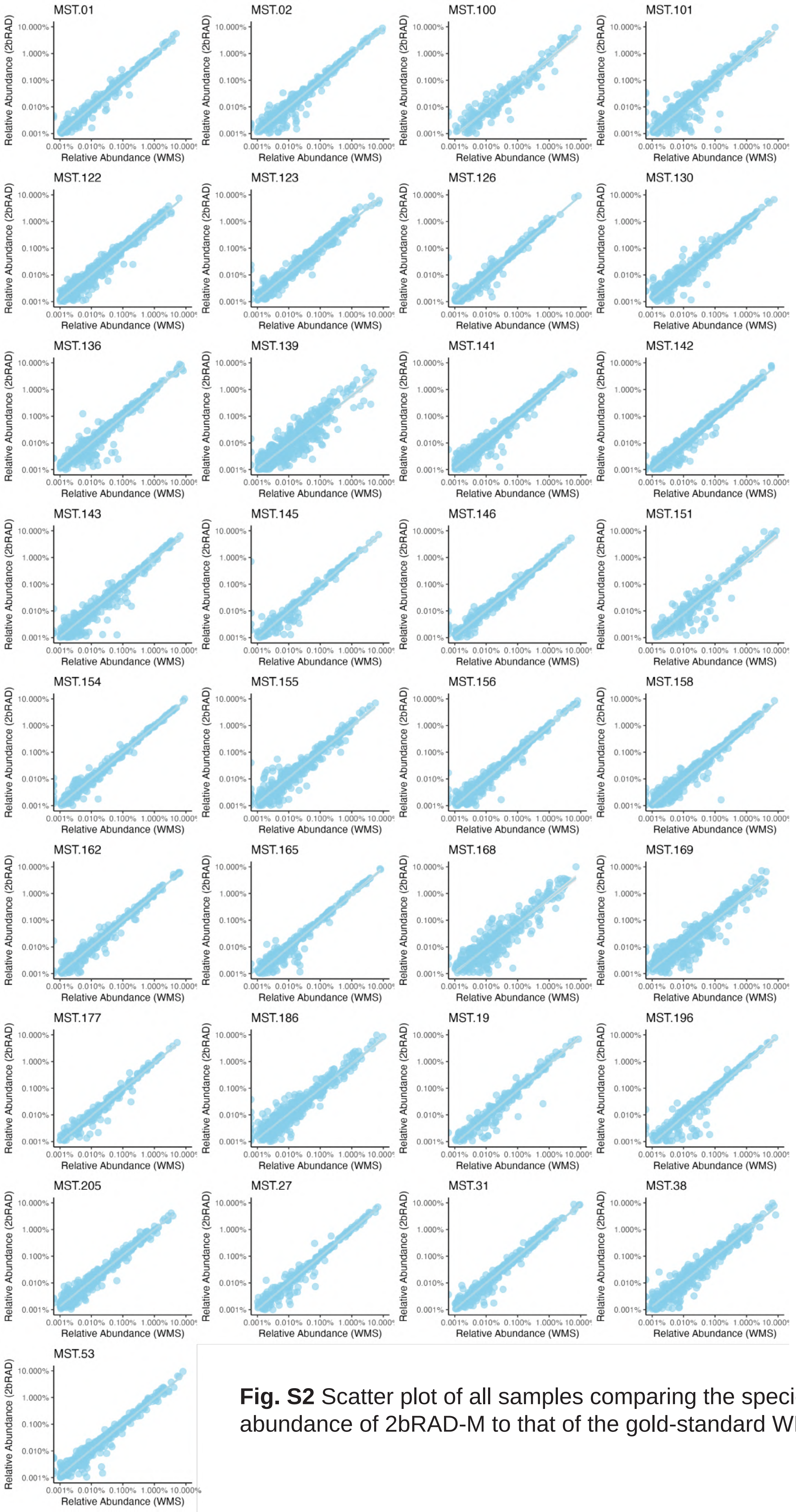

Fig. S2 Scatter plot of all samples comparing the species-level relative abundance of 2bRAD-M to that of the gold-standard WMS method

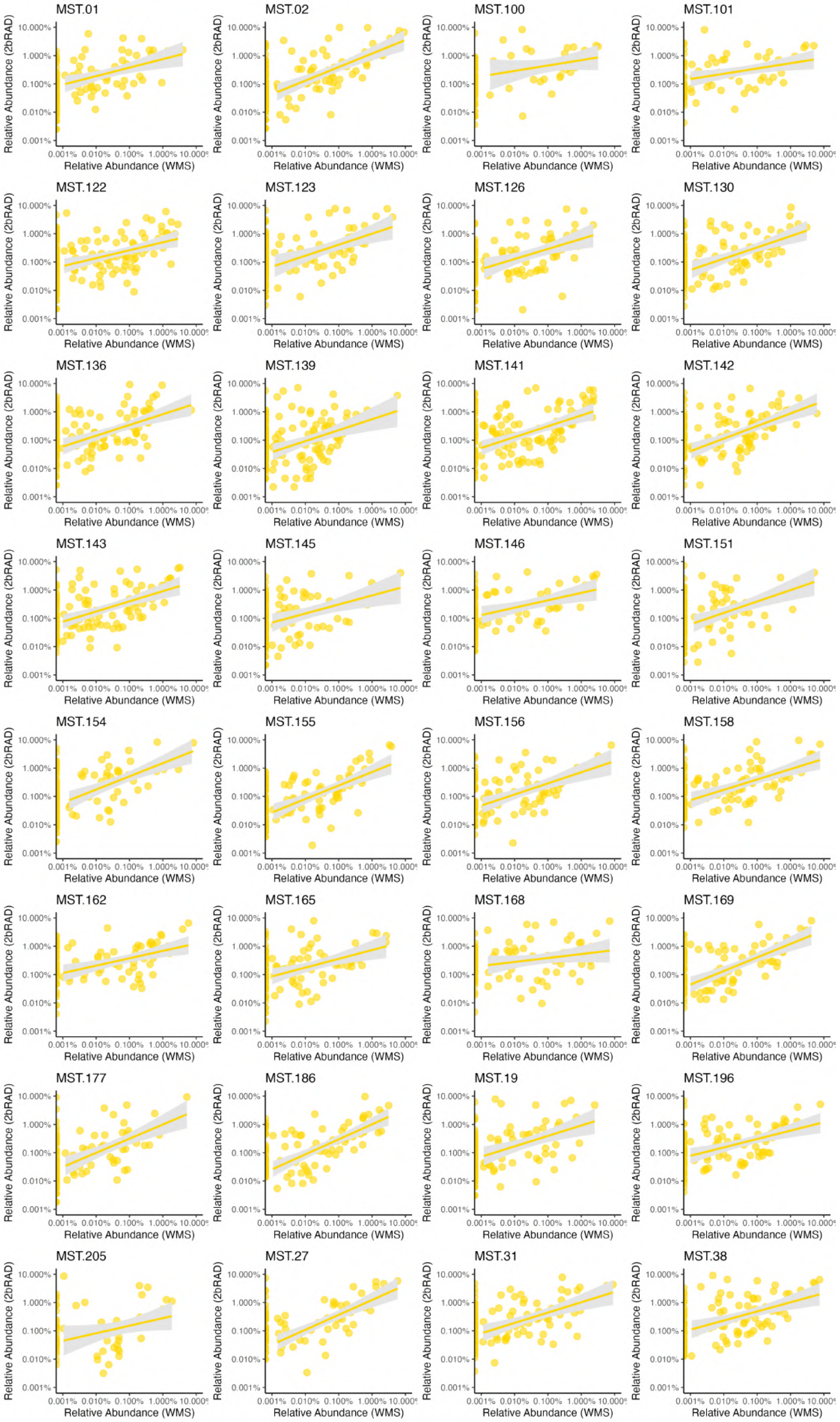

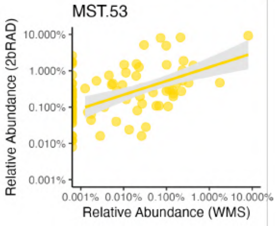

Fig. S3 Scatter plot of all samples comparing the species-level relative abundance of 16S to that of the gold-standard WMS method

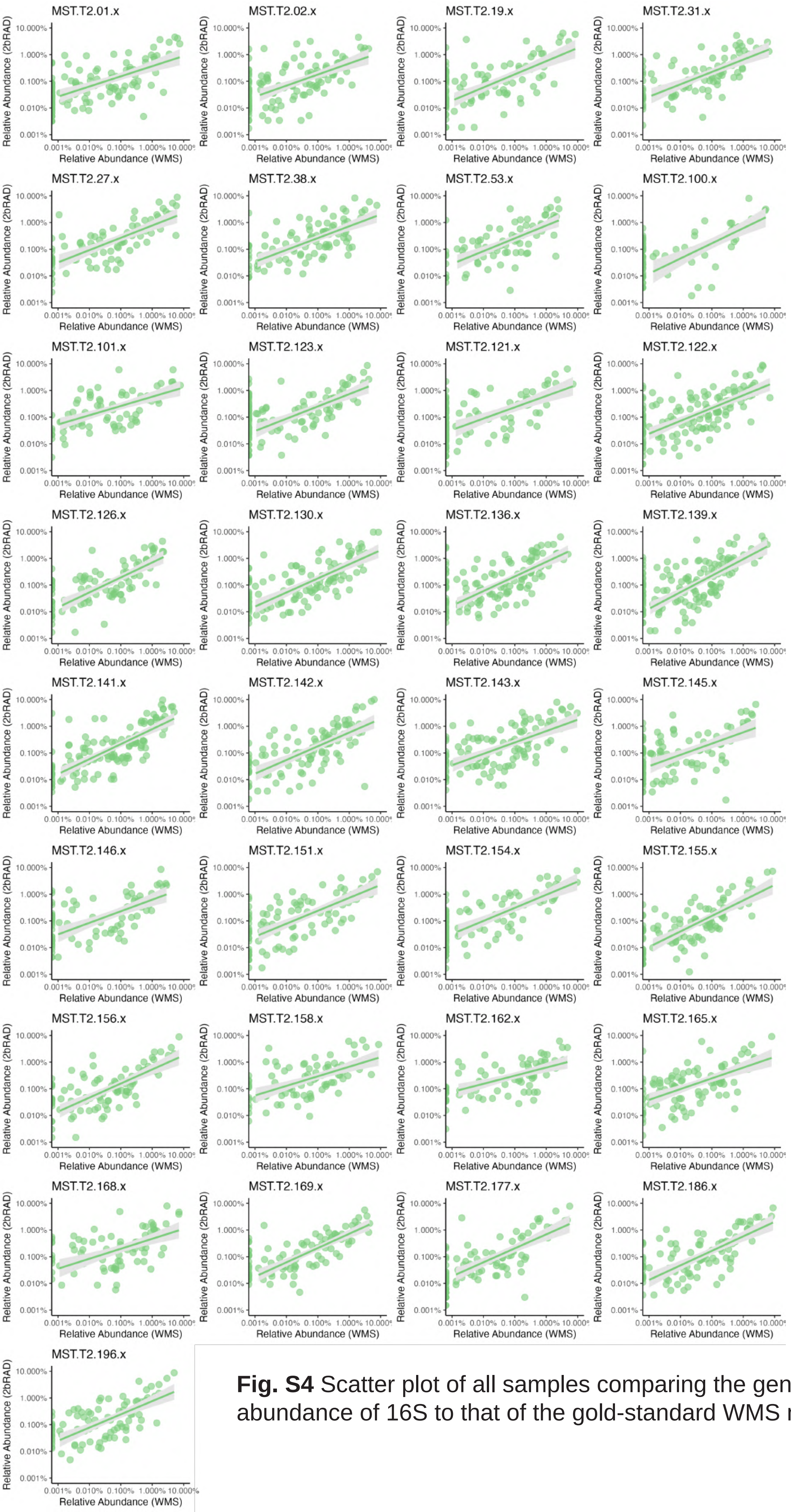

Fig. S4 Scatter plot of all samples comparing the genus-level relative abundance of 16S to that of the gold-standard WMS method
