# Supplementary material for: Unveiling early-life microbial colonization profile through characterizing low-biomass maternal-infant microbiomes by 2bRAD-M
Source: Front Microbiol. 2025 Jan 24;16:1521108. doi: 10.3389/fmicb.2025.1521108 (PMC11802558; doi:10.3389/fmicb.2025.1521108)
Supplement: Supplementary file 2 [file Data_Sheet_2.pdf]

Cetyltrimethylammonium bromide (CTAB) method protocol

1. **Sample Preparation**: Pipette 1000 μL of CTAB lysis buffer into a 2.0 mL EP tube. Add lysozyme and an appropriate amount of sample into the lysis buffer. Place in a 65°C water bath, inverting several times during incubation to ensure thorough lysis of the sample.
2. **Centrifugation and Extraction**: Centrifuge and collect the supernatant. Add phenol (pH 8.0): chloroform: isoamyl alcohol (25:24:1), invert to mix thoroughly, and centrifuge at 12,000 rpm for 10 minutes.
3. **Chloroform Extraction**: Collect the supernatant and add chloroform: isoamyl alcohol (24:1). Invert to mix thoroughly and centrifuge at 12,000 rpm for 10 minutes.
4. **Precipitation**: Transfer the supernatant to a 1.5 mL centrifuge tube, add isopropanol, shake gently, and incubate at -20°C for precipitation.
5. **Washing and Drying**: Centrifuge at 12,000 rpm for 10 minutes. Discard the liquid while ensuring the precipitate remains intact. Wash twice with 1 mL of 75% ethanol. Any remaining liquid can be collected by centrifugation and removed using a pipette tip.
6. **Drying**: Allow the sample to dry in a sterile workbench or at room temperature. (Note: Avoid over-drying DNA samples, as this may make them difficult to dissolve.)
7. **Dissolution**: Add ddH2O to dissolve the DNA sample. If necessary, incubate at 55–60°C for 10 minutes to aid dissolution.
8. **RNA Digestion**: Add 1 μL of RNase A to digest RNA. Incubate at 37°C for 15 minutes.

TIANamp Micro DNA Kit

1. After the breast milk sample has thawed at room temperature, invert to mix well, pipette 200 μL into a 1.5 mL centrifuge tube, and add 200 μL of GA buffer. Vortex to mix well.
2. Add 20 μL of Proteinase K solution and vortex for 10 seconds to mix.
3. Incubate at 56°C until the sample is fully degraded and digested (approximately 1 hour). Invert occasionally during the incubation.
4. After the water bath incubation, add 4 μL of RNase A solution (100 mg/mL), mix well, and let stand at room temperature for 5 minutes.
5. Add 200 μL of buffer GB and 1 μL of Carrier RNA stock solution. Invert to mix thoroughly and incubate at 70°C for 10 minutes, inverting occasionally.
6. Add 200 μL of absolute ethanol. Gently invert to mix the sample and let it stand at room temperature for 5 minutes.

7. Transfer all the solution obtained in the previous step to the CR2 adsorption column (placed in a collection tube). Centrifuge at 12,000 rpm for 30 seconds. Discard the waste liquid and return the CR2 adsorption column to the collection tube.
8. Add 500 μL of buffer GD to the CR2 adsorption column, centrifuge at 12,000 rpm for 30 seconds, discard the waste liquid, and return the CR2 adsorption column to the collection tube.
9. Add 600 μL of PW wash solution to the CR2 adsorption column, centrifuge at 12,000 rpm for 30 seconds, discard the waste liquid, and return the CR2 adsorption column to the collection tube.
10. Repeat the operation from the previous step.
11. Centrifuge at 12,000 rpm for 2 minutes, discard the waste liquid. Open the lid and place the CR2 adsorption column at room temperature for 2-5 minutes.
12. Transfer the CR2 adsorption column to a clean centrifuge tube. Add 30 μL of sterile, enzyme-free water to the center of the adsorption membrane, let it stand at room temperature for 2-5 minutes, then centrifuge at 12,000 rpm for 2 minutes to collect the solution in the centrifuge tube.
13. Quality control: Use Qubit to measure DNA concentration. Pipette 198 μL of working solution into a Qubit tube, add 2 μL of DNA, vortex for 3 seconds, and allow it to stand in the dark for 2 minutes before measuring the concentration. Perform agarose gel electrophoresis to analyze the purity and integrity of the DNA. Use 1% agarose, 125V, for 20 minutes to run the gel.
